# Luteolin-Induced Activation of Mitochondrial BK_Ca_ Channels: Undisclosed Mechanism of Cytoprotection

**DOI:** 10.3390/antiox11101892

**Published:** 2022-09-24

**Authors:** Rafał P. Kampa, Lorenzo Flori, Aleksandra Sęk, Jacopo Spezzini, Simone Brogi, Adam Szewczyk, Vincenzo Calderone, Piotr Bednarczyk, Lara Testai

**Affiliations:** 1 Laboratory of Intracellular Ion Channels, Nencki Institute of Experimental Biology PAS, 02-093 Warsaw, Poland; 2 Department of Pharmacology, Faculty of Pharmacy, University of Pisa, 6 via Bonanno Pisano, 56120 Pisa, Italy; 3 Department of Physics and Biophysics, Institute of Biology, Warsaw University of Life Sciences–SGGW (WULS-SGGW), 159 Nowoursynowska St., 02-776 Warsaw, Poland

**Keywords:** luteolin, cardioprotection, mitoBK_Ca_ channel activation, acute myocardial infarction, apoptosis, necrosis

## Abstract

Luteolin (LUT) is a well-known flavonoid that exhibits a number of beneficial properties. Among these, it shows cardioprotective effects, as confirmed by numerous studies. However, its effect on mitochondrial potassium channels, the activation of which is related to cytoprotection, as well as on heart ischemia/reperfusion (I/R) damage prevention, has not yet been investigated. The large conductance calcium-regulated potassium channel (mitoBK_Ca_) has been identified in both the mitochondria of the vascular endothelial cells, which plays a significant role in the functioning of the cardiovascular system under oxidative stress-related conditions, and in the mitochondria of cardiomyocytes, where it is deeply involved in cardiac protection against I/R injury. Therefore, the aim of this study was to explore the role of the mitoBK_Ca_ channel in luteolin-induced cytoprotection. A number of in vitro, in vivo, ex vivo and in silico studies have confirmed that luteolin activates this channel in the mitochondria of cardiomyocytes and endothelial cells, which in turn leads to the protection of the endothelium and a significant reduction in the extent of damage resulting from myocardial infarction, where this effect was partially abolished by the mitoBK_Ca_ channel blocker paxilline. In conclusion, these results suggest that luteolin has cardioprotective effects, at least in part, through the activation of the mitoBK_Ca_ channel, shedding light on a new putative mechanism of action.

## 1. Introduction 

Numerous studies proving the participation of mitochondrial potassium channels (mitoK) in the life/death processes of cells have been published [1]. One such example is their contribution to the protection of cells against I/R injury at the myocardium [2] and brain level [3,4]. The activation of mitoK channels has been proven to be associated with the induction of cytoprotective processes [5,6,7]. A deeper understanding of these mechanisms offers an opportunity to develop new cytoprotection strategies targeting mitoK channels. However, the molecular, biophysical and pharmacological properties of these channels have still not been fully described [8]. In addition, the previously used synthetic pharmacological substances, considered to be modulators of potassium channel activity, may affect other mitochondrial proteins [9,10].

It has been proven that the activation of the large-conductance calcium-activated mitochondrial potassium channel (mitoBK_Ca_) is associated with the protection of brain cells against damage during stroke [3,4] or cardiac muscle cells, where it alleviates the effects of myocardial infarction [2]. Both the mitochondrial and the plasmalemmal BK_Ca_ channels are perceived as proteins with an important role in cell protection, which has been confirmed by various studies [7,11,12,13,14]. In this regard, the mitoBK_Ca_ channel was identified in the inner mitochondrial membrane (IMM) of the LN229 glioma cells [15]—as well as in the mitochondria of cardiomyocytes [16], dermal fibroblasts [17], skeletal muscle [18], bronchial epithelial [19] and endothelial EA.hy926 cells [20].

The mitoBK_Ca_ channel, like the BK_Ca_ present in the cell membrane, is sensitive to NS1619 and NS11021—known potassium channel activators. Additionally, it was shown that paxilline completely inhibited the activity of this channel [21] and quercetin abolished paxilline inhibition [22]. However, the majority of mitoBK_Ca_ channel modulators exhibit a broad spectrum of side effects [9], including mitochondria-uncoupling properties, inhibition of the respiratory chain, and alteration of calcium ion homeostasis in the cell [23]. Therefore, it seems important to search for new, specific activators of potassium channels, especially natural plant compounds, e.g., from the group of flavonoids. Many data indicate that flavonoid interact with mitochondria in the cell [24,25,26]. Additionally, some flavonoids show cardioprotective properties, such as blood pressure regulation and antioxidant, antiarrhythmic and anti-atherosclerotic properties [27,28], and so it is suspected that the effect may be related to the mitochondrial transport of potassium ions. 

One of the cardioprotective flavonoid is luteolin (3,4,5,7-tetrahydroxy flavone, LUT). This occurs naturally in many species of plants, including celery, carrot, sweet pepper, broccoli, parsley and the green parts of onions [29,30]. It is recognized for its antioxidant, anti-inflammatory, anti-allergic and anti-cancer properties. With regard to its anti-cancer activity, pro-apoptotic- and cell-cycle-inhibiting actions have been demonstrated [31]. In addition, LUT prevents fibrosis in the lungs and liver [32,33]. Of note, LUT is endowed with beneficial effects on the cardiovascular system, and its use has been demonstrated to alleviate myocardial I/R injury as a result of the activation of the antioxidant Nrf2 pathway, as well as helping in the maintenance of mitochondrial integrity. LUT has also been shown to prevent myocardial fibrosis and hypertrophy in diabetic mice, demonstrating a cardioprotective effect [34]. Numerous studies have also confirmed that LUT protects the heart against ischemia/reperfusion (I/R) damage [34,35,36,37], but the role of the mitoBK_Ca_ channel in this phenomenon has not been determined yet.

In addition to the obvious function of the heart in the cardiovascular system, vascular endothelial cells play an important role in its proper functioning. The dysfunction of this cell layer is associated with oxidative stress, vascular dysfunction and atherosclerosis, a condition that increases the risk of cardiovascular events and hypertension. The decrease in the bioavailability of nitric oxide and the rise in the concentration of reactive oxygen species (ROS) are considered to be the main factors involved in endothelial cell dysfunction [38,39].

Considering the above, in this study we investigated the role of the mitoBK_Ca_ channel regulation in the cardioprotective action of LUT, both in vitro in the model of vascular endothelial cells EA.hy926 as well as in vivo and ex vivo in the rat heart. Moreover, through an in silico approach, we suggested LUT binding site within the mitoBK_Ca_ channel.

## 2. Materials and Methods 

### 2.1. Biological and Chemical Materials 

#### 2.1.1. Animal Model 

Experimental procedures (ex vivo and in vivo) were performed in line with the European (EEC Directive 2010/63) and Italian (D.L. March 4, 2014 n.26) legislation (491/2018-PR). Moreover, ARRIVE guidelines were applied. Male Wistar rats (ENVIGO) were housed in cages with unrestricted access to food and water and exposed to a 12 h dark/light cycle. Then, they were subjected to the experimental procedures after reaching the appropriate age (about 3 months) and weight (430–470 g).

#### 2.1.2. Cell Culture 

EA.hy926—human endothelial cell line (Cat. No. ATCC^®^CRL-2922™), originally derived from a human umbilical vein, was used in in vitro studies as an endothelial model [20,40]. Cells were cultured in Dulbecco’s modified Eagle’s medium (1000 mg/L D-glucose) with addition of 10% fetal bovine serum (FBS), 1% l-glutamine, 2% hypoxanthine-aminopterin-thymidine (HAT), as well as 1% penicillin/streptomycin in the atmosphere of 5% CO_2_, 37 °C. The EA.hy926 cells were passaged until they reached approximately 90–95% confluence. Cells between passages 12 and 23 were used in this study.

#### 2.1.3. Chemical Substances

The main substance was luteolin (3,4,5,7-tetrahydroxy flavone, LUT), a natural origin flavonoid (Sigma Aldrich, Milan, Italy). Additionally, the mitoBK_Ca_ channel blocker—paxilline (Sigma Aldrich, Milan, Italy) and opener NS1619 (Sigma Aldrich, Milan, Italy) were used. LUT and potassium channel modulators were dissolved in dimethyl sulfoxide (DMSO, Sigma Aldrich, Milan, Italy) to reach 10 mM concentration and then diluted in buffer solution. Dithiothreitol (DTT; Sigma Aldrich, Milan, Italy) was used to modulate the redox state in the EA.hy926 cells, in particular to obtain a reduced environment in which investigate the role of LUT on mitochondria.

## 3. Experimental Methods

### 3.1. In Vivo Model of Acute Myocardial Infarct 

Male Wistar rats were randomized into three groups (5 animals per group), 2 h before occluding the left coronary artery they were exposed to an intra-peritoneal injection of: vehicle (DMSO 3 mL/kg), LUT (100 mg/kg) and paxilline plus LUT (10 mg/kg of paxilline,100 mg/kg of LUT 20 min later).

Minor changes were applied to experimental procedures previously described [41,42]. Basically, the animals were anesthetized with 70 mg/kg of pentothal sodium (MSD Animal Health) administered through intra-peritoneal injection. Artificial ventilation support system (mod. 7025 Ugo Basile, Comerio, Italy) was set to 70 breaths/min (air volume, 1 mL/100 g body weight).

The chest was opened by a left thoracotomy, then a 6–0 surgical needle was passed 2 mm under the left anterior descending coronary artery was occluded with a 6–0 surgical needle coupled to a small plastic tube. A small plastic tube was placed in front of the coronary artery, then the snare was pulled and fixed in place by clamping the tube with a hemostat, inducing leading to a partial local myocardial ischemia on the left ventricle in the myocardium for 30 min (ischemic period). At the end of the ischemic period, The plastic tube was removed 30 min later from the ligature and the artery was re-opened, allowing reperfusion for 120 min (reperfusion period). An anterior ST segment elevation was observed as a typical event of the ischemic stage, using During monitoring with an electrocardiograph electrocardiographic analyzer (Mindray, PM5000, 2 Biological Instruments, Varese, Italy), increases in T wave and ST segments were observed in the ischemic period; the ST segments returned to normal levels during the reperfusion period.

After the reperfusion period, the heart was removed and a Langendorff apparatus was used to clean up the coronary vessels with Krebs solution (NaHCO_3_ 25 mM, glucose 12 mM, NaCl 118 mM, KCl 5 mM, MgSO_4_ 12 mM, CaCl_2_ 25 mM, KH_2_PO_4_ 12 mM) at 37 °C for 5 min. Only the left ventricle was kept dried and frozen at −20 °C for 20 min, then cut into 2.0 mm sagittal sections. The slices were dipped in a 1% 2,3,5-triphenyltetrazolium chloride (TTC, Sigma-Aldrich, Milan, Italy) solution at 37 °C for 20 min, fixed overnight in a 10% formaldehyde solution and then treated with 3% H_2_O_2_ (Profar^®^) for 10 min before the photographic acquisition. Dehydrogenase enzymes, in the presence of NADH, reacts with TTC to form a formazan derivative, which causes an intense red staining in living cells. The necrotic cells of the infarct area did not show red color, but instead were white or pale pink. The infarct size was evaluated using an image analysis software (Axio Vision Rel 4.8) and expressed as a percentage of the whole ventricle.

In addition, control experiments were carried out in two additional animal groups: SHAM (procedure without induction of ischemia/reperfusion; where each single step of the procedure described above was performed, except for the coronary artery occlusion) and IPC (30-min occlusion described above for the other treatment groups, except for SHAM, was preceded by three cycles of 5 min of ischemia 10 min of reperfusion).

### 3.2. Cardiac Mitochondria Isolation 

Freshly collected heart from a humanely killed animal in accordance with the ethical rules was chopped into small 2/3 mm^3^ pieces in STE buffer (composition: sucrose 250 mM, Tris 5 mM, EGTA 1 mM; pH 7.4) making each procedure steps on ice (4 °C). The tissue scraps were dipped in STE (4°C) and homogenized with Ultra-Turrax (model: IKA, T-18 Basic). Then, a procedure of differential centrifuges was used in order to obtain the isolated mitochondrial component and to maximize its yield. The resulting pellet, containing the mitochondrial fraction, was resuspended in ST buffer (sucrose 250 mM, Tris 5 mM; pH 7.4) and kept on ice until the end of the experimental procedure. The Bradford (Bio-Rad, Hercules, CA, USA) assay tandem spectrophotometric microplate reader (EnSpire, PerkinElmer, Waltham, MA, USA) was used for amount of protein evaluation.

### 3.3. Measurement of Mitochondrial Membrane Potential (ΔΨm)

A potentiometric method consisting of two mini electrodes tandem data acquisition software (Biopac Inc. Goleta, CA, USA) was developed for the evaluation of the mitochondrial membrane potential (∆Ψm) from isolated rat heart mitochondria. The first electrode (WPI, TipTPP, Sarasota, FL, USA) picks up the lipophilic cation tetraphenylphosphonium (TPP^+^), and the second one is a reference electrode (WPI, Sarasota, FL, USA). Then, a mitochondrial suspension (1 mg protein/mL) was made up in swelling buffer (KCl 120 mM, K_2_HPO_4_ 5 mM, Hepes 10 mM, succinic acid 10 mM, MgCl_2_ 2 mM, TPP^+^Cl^−^ 10 μM; pH 7.4) or mannitol buffer (D-mannitol 240 mM, Na_2_HPO_4_ 5 mM, Hepes 10 mM, succinic acid 10 mM, MgCl_2_ 2 mM, ATP 200 μM, TPP^+^Cl^−^ 10 μM; pH 7.4) and was kept under continuous stirring. The ∆Ψm mitochondrial membrane potential value was calculated according to the following experimental equation derived from following the Nernst equation:

(1) $$\Delta \varphi_{m}=60\times \log\frac{\frac{V_{0}{\left[TPP^{+}\right]}_{0}}{{\left[TPP^{+}\right]}_{t}}-V_{t}-K_{0}P}{V_{m}P+K_{i}P}\;\Delta \varphi_{m}=60\times \log\frac{\frac{V_{0}{\left[TPP^{+}\right]}_{0}}{{\left[TPP^{+}\right]}_{t}}-V_{t}-K_{0}P}{V_{m}P+K_{i}P}$$

where: ∆Ψm—mitochondrial membrane potential (mV); *V*_0_—volume of the incubation medium before the addition of mitochondria; *V*_t_—volume of the incubation medium after the addition of the mitochondria; *V*_m_—volume of the mitochondrial matrix (taken as 1 μL/mg protein); [*TPP*^+^]_0_ and [*TPP*^+^]*_t_*—respectively, the TPP^+^ concentrations recorded before addition and at time *t*; *P*—the concentration expressed in mg; *K*_0_ and *K*_i_—external and internal partition coefficients of *TPP*^+^ (14.3 and 7.9 μL/mg, respectively). 4 animals per group were used to ∆Ψm assessment. Data were expressed as mean ± SEM. One-way ANOVA followed by Tukey post hoc test was used to compare groups for statistical differences.

### 3.4. Measurements of the Mitochondrial Ca^2+^ Uptake

Changes in Ca^2+^-concentrations were evaluated by potentiometric method as described above. In this case, however, a reference electrode was coupled to a selective calcium electrode (Ca^2+^ selectivity = 1 × 10^5^ fold) (WPI, Worcester, MA, USA) [41]. Before each experiment, electrodes were calibrated using known concentrations of CaCl_2_. Isolated mitochondria (1 mg protein/mL) were added into the gentle stirring incubation medium and treated with the maximum-used concentration of vehicle (DMSO 0.3%) or LUT (1, 3, 10, and 30 μM). The maximum change of Ca^2+^-concentration in the incubation medium, and then its corresponding storage in the mitochondrial matrix, was measured Mitochondria were isolated from the hearts of three different rats to obtain each result.

### 3.5. Potassium Cations Flow Measurements

The mitoBK_Ca_ channel activation scale was evaluated using a specific Tl^+^-sensitive fluorescent probe (FluxOR, Invitrogen, Monza, Italy), as previously described [42]. After mitochondria isolation (according to *Cardiac mitochondria isolation)* they were incubated with loading buffer (containing the Tl^+^-sensitive probe) for 10 min at room temperature under continuous stirring without light access, in order to allow the Tl^+^-sensitive probe to accumulate in the mitochondrial matrix. Then, residual Tl^+^-sensitive probe was removed from the medium, mitochondria were re-suspended in isolation buffer (400 µL) and kept on ice. Mitochondrial protein concentration was determined with Bradford solution. Mannitol buffer (mannitol 240 mM, Na_2_HPO_4_ 5 mM, HEPES 10 mM, succinic acid 10 mM, MgSO_4_ 2 mM, ATP 200 µM, pH 7.4) was used before the test to further dilute (0.5 mg mitochondrial protein/mL) mitochondrial pool. Later it was placed into a 96-well black plate with clear flat bottom (Corning^®^). LUT (1, 3, 10 and 30 µM) and 30 µM LUT with paxilline (1 and 10 µM added 1 min before LUT). Finally, the entry of Tl^+^ into the mitochondrial matrix, through the potassium channels, was spectrophotometrically monitored for 2 min (λex = 488 nm, λem = 525 nm) with an EnSpire multiplate reader (PerkinElmer, Waltham, MA, USA), after a further addition of Tl_2_SO_4_. The results were analyzed with GraphPad Prism 4.0.

### 3.6. Mitochondria and Mitoplast Preparation for Electrophysiological Studies

For the electrophysiological measurements, newly isolated mitochondria and subsequent mitoplasts were prepared based on differential centrifugation method and hypotonic swelling, respectively, as previously described [20,43]. Mitoplasts were prepared from the endothelial EA.hy926 cells mitochondria through incubation in a hypotonic solution (HEPES 5 mM, CaCl_2_ 100 µM, pH 7.2) for about 1.5 min, and then a hypertonic solution (KCl 750 mM, HEPES 30 mM, and CaCl_2_ 100 µM, pH 7.2) was afterwards added to restore the isotonicity of the medium. For each patch-clamp experiment, fresh mitoplast preparations were used.

### 3.7. Patch-Clamp Experiments

Patch-clamp experiments using endothelial mitoplasts were performed like in previously published studies [14,20,43,44,45]. In simplification, a patch-clamp pipette was filled with an isotonic solution (KCl 150 mM, HEPES 10 mM, and CaCl_2_ 100 µM, pH 7.2). All of the mitoBK_Ca_ channel modulators were added as dilutions in an isotonic solution and applied using a perfusion system. The mitoplasts at the tip of the measuring glass pipette were transferred into the openings of a multibarrel piping system in which their outer faces were rinsed with the test solutions. The current-time traces of the experiments were recorded, and then the open probability (P_o_) of the channels was determined using the single-channel search mode of Clampfit 10.7 software. Results are presented as the mean ± SD which were obtained from at least three to six independent experiments conducted in triplicates.

### 3.8. Cell Respiration Measurements

Cells for measuring oxygen consumption (cellular respiration), under the influence of the tested flavonoid, were grown in complete cell culturing medium. Experiments were performed using an oxygraph (O2k-respirometer, Oroboros Instruments^®^). After starting the device, its electrodes in individual chambers were rinsed and stabilized (approx. 30 min), first with water, and then with the clear DMEM (cDMEM—DMEM without the addition of FBS, HAT, glutamine, penicillin and streptomycin) at 37 °C. Subsequently, cells were detached from the cell culture flasks with trypsin, which was then centrifuged for 5 min (300 × *g*). The cell pellet was resuspended in cDMEM without additives (37 °C). Cells were counted, resuspended in 2.1 mL of cDMEM, and transferred to oxygraph chambers, 2 million cells per chamber, which were then sealed without leaving any air bubbles inside. After another stabilization of the measurement system for 20 min, the titration of LUT was started. The experiment was ended by decoupling the mitochondria by adding FCCP (500 nM) to the chamber. The results obtained were compared with non-treated cells respiration. Additional tests of respiration were conducted with cells preincubated with 0.5 mM DTT (in case of reduced conditions as in patch-clamp experiments) per 1 h before detaching them from the bottom of the flask. Then, the above-described procedure was followed.

### 3.9. Apoptosis/Necrosis Assay

RealTime-Glo™ Annexin V Apoptosis and Necrosis Assay (Promega, Madison, WI, USA) kit, measuring apoptosis and necrosis singlas, was used to check the toxicity of LUT at different concentrations (3, 10, 30 μM). EA.hy926 cells were sown on a 96-well assay black plate (Corning Incorporated Costar^®^) at 8500 per well, and then after 48 h of culture in complete cell culture medium, a test was carried out. Before starting the measurements, medium was changed, and to 100 μL of fresh medium, freshly prepared, according to the manufacturer’s instructions, the RealTime-Glo™ test in a 1:1 ratio was added. Measurements were performed with an Infinite m200 pro plate reader (Tecan^®^, Grödig, Austria) at designated points in time: 0, 1, 3, 7, 9, 11, 13 and 24 h. As a positive control, the combination of TNF-α (tumor necrosis factor α) 1 ng/mL and cycloheximide 0.05 μg/mL (TNF/CHX) [14,46] was used. This mix is widely used to induce in the cells an oxidative stress and apoptotic damage [47].

### 3.10. Molecular Modeling Studies

For docking of LUT, the K^+^ Slo1 channel was used (PDB ID 6V3G) [48]. The structure of LUT was obtained from the PubChem online database CID 5280445 and optimized before docking using the LigPrep module available in Schrödinger suite (LigPrep release 2020, Schrödinger, LLC, New York, NY, USA, 2020) using the OPLS3e force field. Molecular docking of LUT was performed using the Induced Fit Docking (IFD) (IFD release 2020, Schrödinger, LLC, New York, NY, USA, 2020) with the use of the implicit membrane setting, employing the OPLS3e force field. The channel was prepared by protein preparation wizard protocol. While the binding site analysis was conducted using the application SiteMap (SiteMap release 2020, Schrödinger, LLC, New York, NY, USA, 2020) The cubic box for ligand docking was centered on the residues Met285, Thr287, Gly311 and Phe315 with a 10 Å box size similar to previously published quercetin/paxilline docking [22]. LUT has been docked flexibly with a maximum pose number equal to 20. Then, the refinement of residues located within 5 Å of ligand poses was performed with side chain optimization, and the ligands were redocked with extra precision (XP) using the Glide protocol. 

### 3.11. Statistical Analysis 

ANOVA (One- or two-way) with Tukey post hoc test were used to compare the means of three or more treatment conditions using GraphPad Prism 5 (GraphPad Software). For all tests, a *p*-value was considered to be significant at *p* < 0.05 (*), *p* < 0.01 (**), *p* < 0.001 (***), or *p* < 0.0001 (****); or non-significant (ns). Each of performed experiments were carried out in at least three independent repetitions (each of them also internally at least 3 times repeated).

## 4. Results

### 4.1. In Vivo and Ex Vivo Studies 

#### 4.1.1. In Vivo Acute Myocardial Infarction and LUT Action

**Figure 1:**
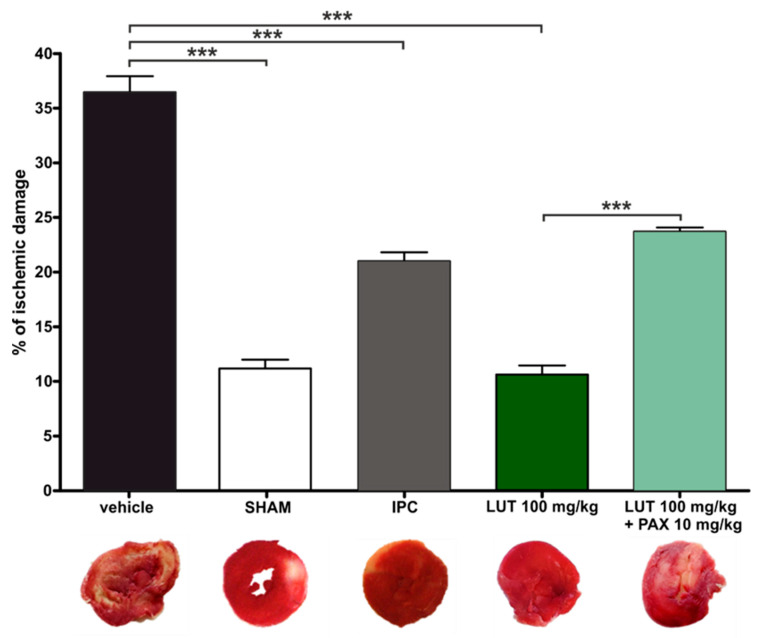
Morphometric quantification of ischemia/reperfusion-induced injury observed in ventricular slices of rat hearts, after acute myocardial infarction in vivo. Graph shows % of ischemic damage in comparison to whole slice area for vehicle (3 mL/kg DMSO), SHAM, IPC and after treatment with LUT (100 mg/kg) and LUT with paxilline (100 mg/kg + 10 mg/kg). Additionally, the figure shows exemplary photographs of cross-sections through the left ventricle with a stained ischemic zone in the given experimental conditions. The level of statistical significance was determined by one-way ANOVA with Tukey post hoc test, *n* = 3, *p* < 0.001 (***)).

According to the procedure described in the Materials and Methods, 100 mg/kg LUT was administered intraperitoneally 2 h before the induction of myocardial infarction (occlusion of the left ventricular artery). The occlusion lasted 30 min (ischemia), followed by a 2 h reperfusion and collection of the heart, its washing, freezing, cutting into 2 mm slices and staining of the ischemic damage. Digitally fixed slices of the left ventricle were analyzed and it turned out that in the case of LUT, the ischemic damage was 10.5% ± 0.8%. Exactly the same experiments were also carried out on rats which were administered 10 mg/kg paxilline intraperitoneally 20 min before LUT injection, as well as 3 mL/kg DMSO (the maximum volume of vehicle used for LUT and paxilline). The ischemic damage in the paxilline plus LUT treatment was greater than those in the luteolin-treated group and reached 23.7% ± 0.5%. The greatest damage to the heart was reported in the group of rats treated with vehicle—DMSO (36.5% ± 1.5%), which means that the vehicle was not responsible for the observed protective effect. For SHAM and IPC groups, the ischemic damage was 11.0% ± 2.0% and 21.0% ± 2.0%, respectively. Obtained results are presented in Figure 1 and suggest a significant role of the mitoBK_Ca_ channel activation by LUT in the protection of the myocardium against ischemia/reperfusion injury.

#### 4.1.2. Ex Vivo Changes in Membrane Potential in Cardiac-Isolated Mitochondria

**Figure 2:**
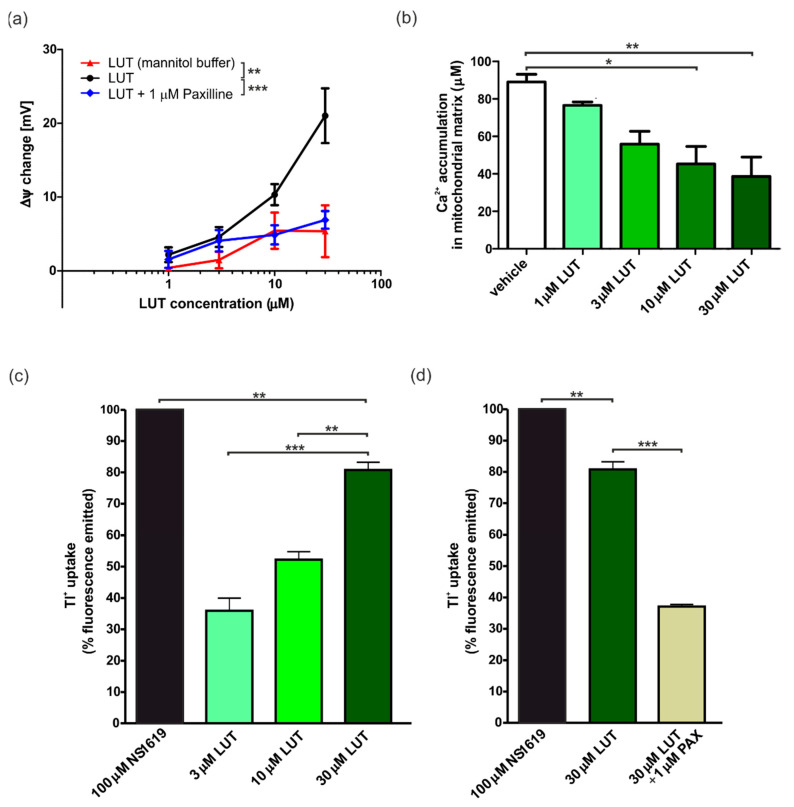
Ex vivo determination of mitoBK_Ca_ channels activation properties by LUT. (**a**) Changes in ΔΨ in the presence of LUT. The increasing mitochondrial depolarization is shown for LUT 1, 3, 10 and 30 µM. The blue line corresponding to results obtained in conditions where 1 µM paxilline were added 2 min before LUT and the red line to results obtained for LUT in mannitol buffer (potassium-free) for the same flavonoid concentrations. The level of statistical significance between these two conditions was determined by one-way ANOVA with Tukey’s test for *n* = 3 (*p* < 0.01 (**); *p* < 0.001 (***)). (**b**) Impact of LUT on calcium ions accumulation in the cardiac mitochondrial matrix. The graph shows the µM concentration of calcium uptaken by mitochondria in probes with 1, 3, 10 and 30 µM LUT and with vehicle (0.3% DMSO; the maximum concentration of the vehicle used). The level of statistical significance in comparison to 0.3% DMSO was determined by one-way ANOVA with Tukey’s test for *n* = 3 (*p* < 0.05 (*), *p* < 0.01 (**)). (**c**) Changes of K^+^ flux in the presence of LUT measured by Tl^+^ fluorescent probe. Graph shows the % fluorescence emitted vs. 100 µM NS1619 (100%) as maximal mitoBK_Ca_ channel activation and for 3, 10 and 30 µM LUT. The level of statistical significance was determined using one-way ANOVA with Tukey’s test for *n* = 4 (*p* < 0.01 (**), *p* < 0.001 (***)). (**d**) Inhibition of mitoBK_Ca_ channels by paxilline measured by Tl^+^ fluorescent probe. Graph shows the activation of the channel by 30 µM LUT vs. NS1719 and its inhibition in probe 30 µM LUT with 1 µM paxilline (PAX). The level of statistical significance was determined using one-way ANOVA with Tukey’s test for *n* = 4 (*p* < 0.01 (**), *p* < 0.001 (***)).

To evaluate proper involvement of the mitoBK_Ca_ channels, the effect of LUT on changing the potential of the IMM was determined using a potentiometric approach, by means a TPP^+^-sensitive electrode, on isolated mitochondria from rat hearts. A concentration-increasing administration of LUT, showed a progressive change in potential, indicative of depolarization. LUT 1 µM induced a change in ΔΨ of 2.2 ± 1.0 mV, 3 µM of 4.6 ± 14 mV, 10 µM of 10.3 ± 1.4 mV and finally LUT 30 µM induced a depolarization of 21.0 ± 3.7 mV. In order to determine the possible participation of mitochondrial potassium channels in the observed event of depolarization, these experiments were performed once again, but in a potassium-free buffer (mannitol buffer). No significant changes were observed this time (ΔΨ 1 µM: 0.4 ± 0.4 mV; 3 µM: 1.5 ± 1.1 mV; 10 µM: 5.4 ± 2.5 mV; 30 µM: 5.4 ± 3.5 mV). Addition of 1 µM paxilline significantly reduced the observed depolarization effect (change in ΔΨ for LUT: 1 µM: 1.6 ± 0.6 mV; 3 µM: 4.1 ± 0.7 mV; 10 µM: 4.9 ± 0.7 mV; 30 µM: 6.9 ± 0.6 mV), almost to the level observed in the mannitol buffer. The results are presented in Figure 2a.

#### 4.1.3. Ex Vivo Calcium Ions Uptake into Cardiac Mitochondria

Another attempt was to determine the value of calcium ions uptake in the presence of LUT. Indeed, in the case of mitoK channel activators, an inverse relationship between the depolarization and increasing concentration of activators was observed. In this case, the highest the mitoK channel activation, followed by mitochondrial depolarization, results to the lowest calcium ions uptake [49,50]. After the mitochondria were isolated from the rat heart, measurements were started with the use of a calcium ion selective electrode, and then results collected. For vehicle (0.3% DMSO) the value of Ca^2+^ uptake was 89.1 ± 4.1 µM. Conversely, a concentration-dependent decrease in the calcium ions accumulation in mitochondria was recorded after the addition of LUT. In particular, with 30 µM of LUT the value raised 38.6 ± 10.3 µM, for 10 µM LUT 45.3 ± 9.4 µM, and for 3 and 1 µM LUT 55.9 ± 6.9 µM and 76.6 ± 1.9 µM, respectively. The detailed results are presented in Figure 2b.

#### 4.1.4. Ex Vivo Potassium Ions Flow through mitoBK_Ca_ Channels in Cardiac Mitochondria

In the freshly isolated rat heart mitochondria, the flow of potassium ions was further determined using thallium (Tl^+^) membrane permeability labeling and spectrophotometric method. NS1619, a well-known mitoBK_Ca_ channel activator, produced a high Tl^+^ flow, that we used as 100%. The results indicated that as the concentration of LUT increased (3, 10 and 30 µM), the uptake of Tl^+^ ions increased (respectively: 35.9% ± 6.9%; 52.3% ± 4.3%; 80.9% ± 6.0%), suggesting a significant increase in the flow of potassium ions, and thus confirming the activation of mitoK channels. The use of the selective mitoBK_Ca_ channel blocker—paxilline 1 µM decreased the influx of Tl^+^ into the mitochondrial matrix (35.1% ± 1.0%). This confirms both the participation of this type of channel in the observed phenomenon and its activation by LUT with an increasing concentration. The results shown in Figure 2c,d represent the cumulative values after subtracting the baseline value, which was the value for 1% DMSO (maximal vehicle concentration used). 

### 4.2. In Vitro Model of Endothelial Cells: EA.hy926 

#### 4.2.1. Regulation of mitoBK_Ca_ Activity by LUT under Different Redox Conditions

**Figure 3:**
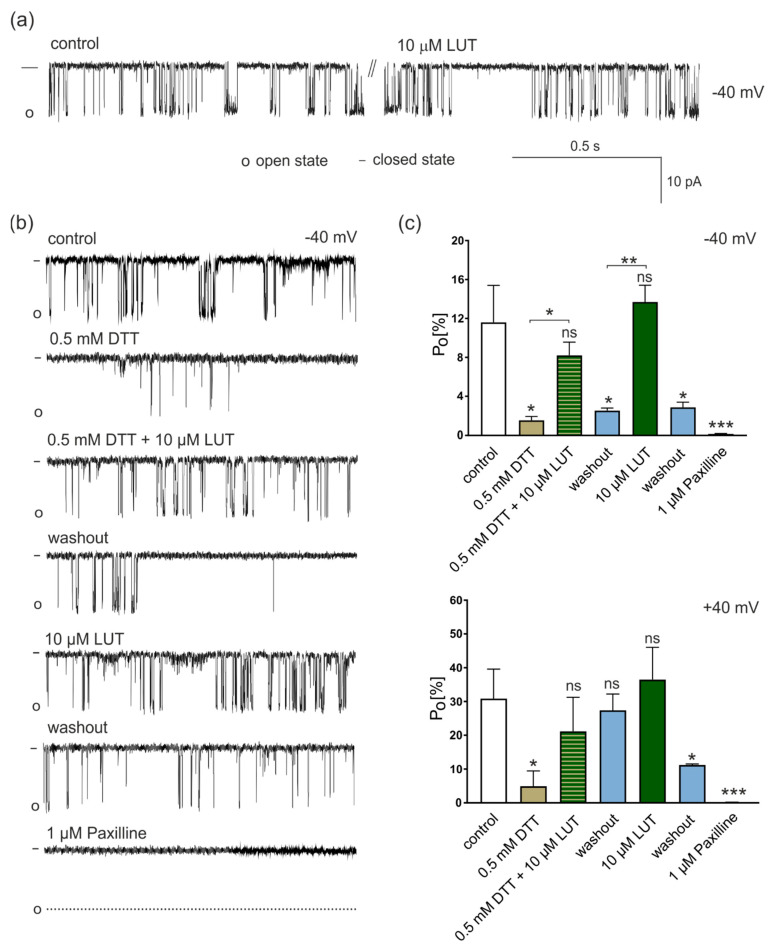
Regulation of mitoBK_Ca_ channel activity by LUT. (**a**) Effect of LUT on the activity of the mitoBK_Ca_ channel under control conditions. The figure shows the activity of the mitoBK_Ca_ channel present in the inner mitochondrial membrane of vascular endothelial cells and after the administration of 10 μM LUT. The presented records were obtained −40 mV in a symmetrical buffer of 150/150 mM KCl, in the presence of 100 μM Ca^2+^, pH = 7.2. “-” means the closed state of the channel; “o” means the open state of the channel. (**b**) Regulation of mitoBK_Ca_ channel by LUT in reduced conditions. Exemplificative recordings of channel activity under control conditions, then reduced conditions of 0.5 mM DTT, reduced conditions of 0.5 mM DTT in the presence of 10 μM LUT, after washout to control conditions, after administration of 10 μM LUT without DTT, after the second washout and under the influence of 1 μM paxilline. The measurements were made at +40 mV and −40 mV in a symmetrical buffer of 150/150 mM KCl, in the presence of 100 μM Ca^2+^, pH = 7.2. “-” means the closed state of the channel; “o” means the open state of the channel. (**c**) Analysis of the probability of channel openings (P_o_). P_o_ evaluation for the individual stages of the experiments in part (**b**) with the determination of the levels of statistical significance (one-way ANOVA with Tukey’s test), *n* = 4 (*p* < 0.05 (*); *p* < 0.01 (**); *p* < 0.001 (***); *p* > 0.05 (ns)).

Taking into account the functioning of the whole cardiovascular system, we also wanted to check the influence of LUT on mitoBK_Ca_ in a model of endothelial cells. Indeed, recent reports indicate that the mitoBK_Ca_ is the channel most often observed in mitochondria of the EA.hy926 cell line, providing then a reliable model for testing the effects of putative activators of mitochondrial potassium channels. In the first stage of the research, using the patch-clamp technique, the effect of LUT on the activity of the mitoBK_Ca_ was determined. Biophysical and pharmacological characterization with well-known modulators of the mitoBK_Ca_ channel has previously been carried out in this cell line [14,20,51]. The experiments were performed in a symmetrical system of concentrations of 150/150 mM KCl, in the presence of 100 μM Ca^2+^, pH = 7.2. After administration of 10 μM LUT, no significant changes in the mitoBK_Ca_ channel activity were observed at the −40 mV voltage (same for +40 mV—data not shown). Sample fragments of recordings under control conditions and then after the administration of 10 μM LUT are presented in Figure 3a.

Due to the lack of influence of LUT on the activity of the mitoBK_Ca_ channel under the control conditions, we decided to change the redox state of experimental conditions. Although many mechanisms are involved in ensuring that the redox processes in the mitochondrial matrix are stable, conditions in the mitochondria due to the presence of, among others, ROS are sometimes more reduced and sometimes slightly more oxidized. In order to evaluate if redox conditions could influence the activity of the mitoBK_Ca_ channels and then the activation properties of LUT, we carried out a series of experiments using DTT, a compound used to protect the thiol group against oxidation or reduction in disulfide bridges [52], e.g., between the sulfur atoms of cysteine residues occurring in the amino acid sequence of the channel pore-forming domains [53]. The results of the experiments performed in the presence of DTT are shown in Figure 3b,c. 

In particular, at −40 mV after addition of 0.5 mM DTT probability of openings (P_o_) of mitoBK_Ca_ decreased from 11.6% ± 3.8% to 1.5% ± 0.4%. Then, 10 μM LUT *plus* DTT increased the channel activity to 8.2% ± 1.4%, and after washout, the probability of mitoBK_Ca_ channel openings decreased again to 2.5% ± 0.2%. 10 μM LUT administered at the background of the control solution without DTT increased P_o_ to 13.7% ± 1.7%, which dropped again to 2.8% ± 0.5% after washout. In the final stage of the experiment, the administration of 1 μM paxilline resulted in complete closure of the mitoBK_Ca_ channel.

The mitoBK_Ca_ activity was slightly different at positive voltage (+40 mV). In the control, the probability of channel opening was on average 30.8% ± 8.3%, and after the administration of 0.5 mM DTT it decreased to 4.9% ± 5.3%. After administration of DTT *plus* 10 μM LUT, it increased to 21% ± 10.2%. Then, as a result of washout to the control conditions, P_o_ increased to 27% ± 5.3%, and administration of 10 µM LUT without DTT caused an increase in P_o_ of the mitoBK_Ca_ channel to the value of 36.5% ± 11.1%. Subsequent washout reduced the channel activity to 11.2% ± 0.2%, and 1 μM paxilline blocked it. Therefore, 10 µM LUT did not change the mitoBK_Ca_ channel activity under control conditions. Conversely, under reduced by DTT conditions, it activated it at both positive and negative voltages, however, at +40 mV the results showed no statistical significance. These results suggested a role of LUT in the prevention against pro-oxidant conditions, such as myocardial ischemic events or vascular dysfunctions, in which mitoBK_Ca_ channels can be crucial.

#### 4.2.2. Impact of Luteolin on Oxygen Consumption 

**Figure 4:**
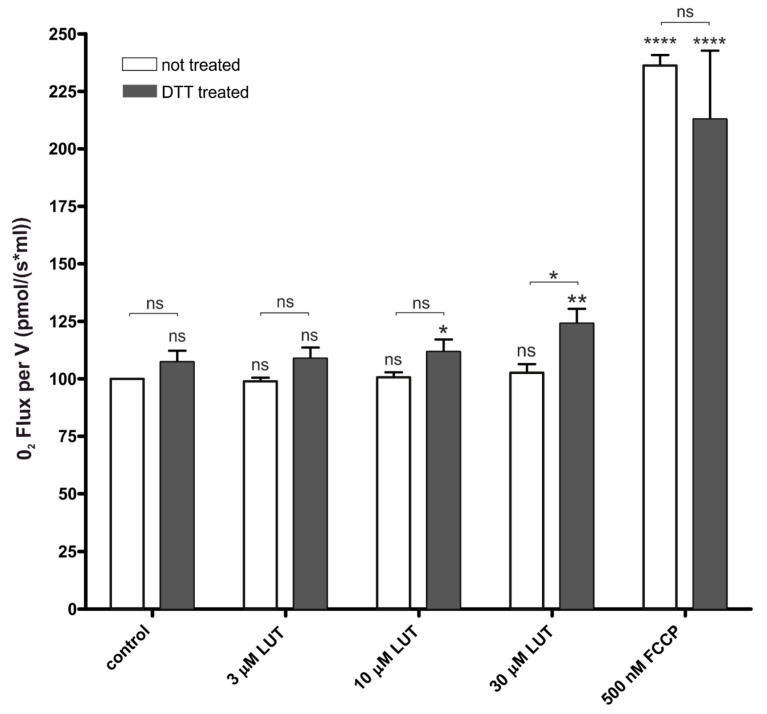
Changes in oxygen consumption in control and DTT-treated endothelial cells under the influence of LUT. A comparison of the data for the effect of 3, 10 and 30 µM LUT on cell respiration in the control group and those treated with 0.5 mM DTT is presented. Statistical significance levels, with respect to non-treated control (above the column) and between probes, were determined using the two-way ANOVA with Tukey’s test for *n* = 3 (*p* < 0.05 (*), *p* < 0.01 (**), *p* <0.0001 (****), *p* > 0.5 (ns)).

Mitochondrial ion channels modulators have been described as factors that increase the respiration level [14,44,45,54]. On the basis of this concept, NS11021 and NS1619 [55], known as synthetic mitoBK_Ca_ activators, as well as naturally derived activators such as naringenin [14,44], enhance mitochondrial respiration. Based on the results of patch clamp experiments under basic conditions, LUT did not activate the channel. This translated into the results of mitochondrial respiration, where no changes were observed after the administration of the entire spectrum of tested LUT concentrations, in relation to the control. However, when LUT was administered to the cells preincubated with 0.5 mM DTT, a significant increase in mitochondrial respiration with 10 µM and 30 µM LUT was observed (see Figure 4). In this case, the results also correspond to the patch-clamp data, where under reduced conditions, LUT showed mitoBK_Ca_ channel activating properties. These results may suggest commitment of mitoBK_Ca_ channel activation by LUT in the observed effect.

#### 4.2.3. Anti-Apoptotic, Cytoprotective Effects of LUT 

**Figure 5:**
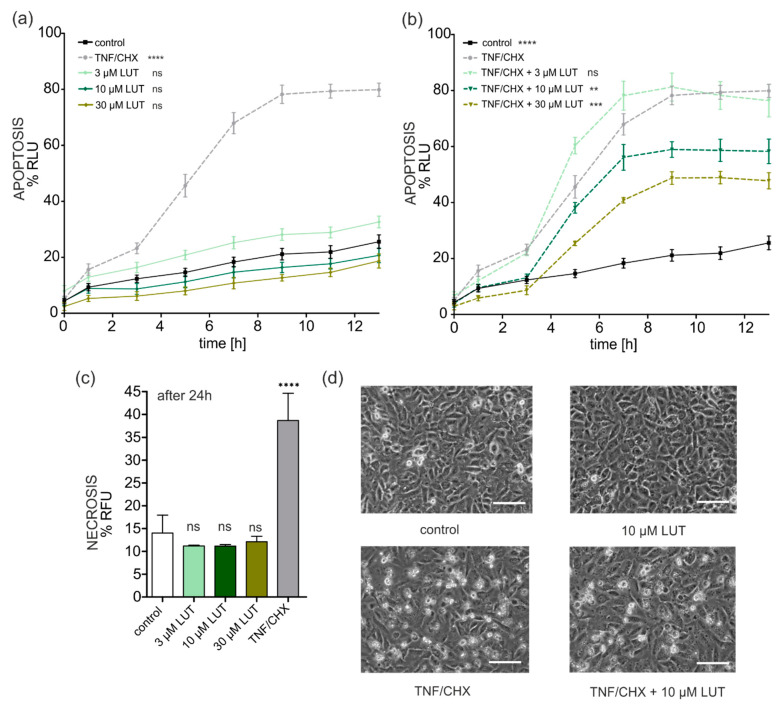
Protective effects of LUT against damage caused by TNF-α with cycloheximide. (**a**) Effect of LUT on apoptosis of EA.hy926 cells. The results of experiments under control conditions, after the addition of 3, 10 and 30 µM LUT in relation to the inducer of apoptosis in endothelial cells—TNF/CHX, are presented (% RLU-relative luminescence units; corresponded to the signal of apoptosis). Statistical significance levels relative to control were determined using a two-way ANOVA with Tukey’s test for *n* = 3 (*p* < 0.0001 (****); *p* > 0.05 (ns)). (**b**) Protective effect of LUT on damage by TNF/CHX. The graph shows the change in luminescence over time (induction of apoptosis) under control conditions and in cells incubated with 3, 10 and 30 µM LUT one hour before the addition of dTNF/CHX, and in damaged TNF/CHX cells. Statistical significance levels relative to the damage ddprobe were determined using the two-way ANOVA with Tukey’s test for *n* = 3 (*p* < 0.01 (**); *p* < 0.001 (***); *p* < 0.0001 (****); *p* > 0.05 (ns)). (**c**) Effect of LUT on induction of necrosis in EA.hy926 cells after 24 h. The graph shows the results obtained under control conditions, 3, 10 and 30 μM of LUT and the damage factor TNF/CHX. The level of statistical significance relative to the control was determined by one-way ANOVA with Tukey’s test for *n* = 3 (*p* < 0.0001 (****); *p* > 0.05 (ns)). (**d**) Photographs showing the observed protective effect of 10 μM LUT against TNF/CHX damage in vascular endothelial cells, taken after 24 h. Pictures of control cells treated with 10 μM LUT, damaged by TNF/CHX and pre-incubated with 10 μM LUT before the administration of the damaging factor (TNF/CHX) are presented. The white horizontal line in the lower right corner is 200 µm.

LUT toxicity to EA.hy926 cells was determined using an apoptosis and necrosis detection assay. The results for apoptosis are shown in Figure 5a. LUT at concentrations of 3, 10 and 30 µM did not induce apoptosis as compared to cells damaged by the treatment with TNF/CHX (1 ng/mL/0.05 µg/mL). The results were superimposable to those obtained in the untreated control.

On the other hand, LUT emerged as a protective agent against the oxidative stress and apoptosis caused by the combination of TNF/CHX. Indeed, one hour before adding the damaging factors to the cells, they were incubated with LUT at concentrations of 3, 10 and 30 μM. The results regarding protection against the induction of oxidative stress and apoptosis are shown in Figure 5b. It was found that 3 µM LUT did not protect endothelial cells from TNF/CHX-induced damage, but a significant protection was observed with 10 and 30 µM LUT, indeed the apoptotic signal was, after 8 h, more than 20% and 30% lower (respectively).

Additionally, the potential protective properties of LUT against necrotic cell death were determined by measuring the fluorescence signals 24 h after the start of the assays. The results are presented in Figure 5c. LUT at tested concentrations did not induce necrosis even after 24 h. However, no statistically significant differences in protection against necrotic processes were observed, therefore LUT did not protect EA.hy926 cells from TNF/CHX induced necrosis (data not shown).

Representative photographs of the protective effect of 10 µM LUT are shown in Figure 5d (less damaged cells in comparison to TNF/CHX treated cells).

### 4.3. In Silico Studies with LUT and the mitoBK_Ca_ Channel

**Figure 6:**
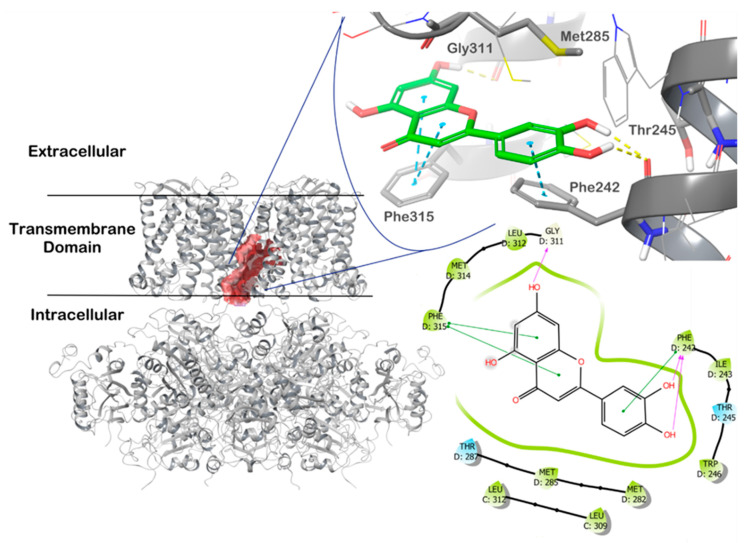
Molecular docking of LUT in the BK_Ca_ channel pore (PDB ID 6V3G). The figure shows the cross-section of the mitoBK_Ca_ channel with the identified binding site, represented as red surface, and the LUT molecule docked at the most likely binding site). The picture was generated by Maestro (Maestro release 2020-3).

In order to gain insight into the possible mechanism of LUT involving the interaction with the mitoBK_Ca_ channel, in silico molecular modeling studies were also carried out, employing an IFD protocol as previously reported [22]. These studies highlighted the possibility that LUT can strongly interact with the channel as proposed by experimental studies, providing a detailed mode of interaction at the molecular level (Figure 6). In particular, after the identification of the most probable binding site within the transmembrane domain of the channel by the application SiteMap, the IFD calculation was restricted to that region. According to the calculation, this region is composed of the residues Met285, Thr287, Gly311, and Phe315. The IFD results showed a strong network of interactions of LUT within the selected binding site. LUT can target Thr245 by two H-bonds and the backbone of Gly311 with a H-bond, while a double π-π stacking was observed with Phe315, and a single π-π stacking was detected with Phe 242. This binding mode accounted for a docking score of −8.403 kcal/mol. These results are in perfect agreement with the previous computational studies conducted on a strongly related compound, quercetin, for which a comparable docking score and binding mode was found applying the same protocol [22].

## 5. Discussion

This work provides a comprehensive look at the protective effect of luteolin (LUT) in the cardiovascular system, indicating that supplementation with this flavonoid could contribute to limiting the oxidative damage in the vascular endothelium at the first step, which can prevent the development of atherosclerosis and further contribute to the protection of the heart against infarction damage. A series of experiments indicate the existence of a new mechanism of cardioprotection for LUT. It is known that luteolin manifests these properties and also protects the heart against myocardial infarction, which has been proven previously [35,56,57,58,59], but the contribution to and role of mitoBK_Ca_ channel in this phenomenon has not been described yet. 

The role of LUT in the regulation of the plasmalemmal potassium channels activity has been described in many studies [34,60,61]; however, there are no reports showing its influence on the mitoK channels. The results presented in this study concerning the vascular endothelium and rat heart are therefore the first in this area. In the course of this research, no unequivocal effect of LUT on the mitoBK_Ca_ channel of EA.hy926 endothelial cells was proven under control conditions, i.e., in the presence of 100 μM Ca^2+^; in this case, the effect of LUT was determined to be neutral. Due to the lack of regulation of the mitoBK_Ca_ channel by LUT, experiments in a reduced environment were conducted. For this purpose, dithiothreitol (DTT) was used. DTT is a compound that reduces proteins, including channel proteins [62,63,64]. It has been proved that DTT inhibits the activity of the plasmalemmal BK_Ca_ channel, which means that under reduced conditions the potassium current does not flow through the channel [65]. It is also worth mentioning that the effect of DTT applied to whole cells leads to the depolarization of the IMM and an increase in the synthesis of ROS [66]. It is known that ROS are mainly formed in the mitochondria [67] as well as the high levels of ROS can lead to mitochondrial dysfunction [68,69]. Therefore, LUT activates mitoBK_Ca_ channels in cells with high ROS synthesis caused by DTT exposition. Moreover, in the model of isolated mitochondria from rat hearts, we proved that LUT increases the flow of potassium ions, which also suggests the activation of potassium channels [42,49]. The trial with paxilline that limited this phenomenon shows an undoubted involvement of the mitoBK_Ca_ channel. LUT is therefore another flavonoid, beside naringenin [14,17,70] and quercetin [51], which activates the mitoBK_Ca_ channel. The mere participation of LUT in the activation of potassium channels in mitochondria isolated from the rat heart was also confirmed by the calcium ions uptake studies, where increasing LUT concentrations reduced the uptake levels of calcium. As mentioned before, the higher mitoK channel activation, the lower the calcium uptake value is observed [49,50]. Considering the above, the obtained results proving the activation of the mitoBK_Ca_ channel by LUT in endothelial cells, under reduced conditions, may suggest its protective effect on mitochondria, against their dysfunction caused by the very high concentration of ROS. Moreover, in addition to activating mitoBK_Ca_ in the endothelium, LUT can activate this channel in rat heart mitochondria.

The activation of mitoK channels changes the potential of the IMM. The influx of potassium ions into the negatively charged mitochondrial matrix depolarizes the mitochondria [71,72,73]. The subtle regulation of the activity of mitoK channels is necessary to avoid abrupt changes in the mitochondrial potential [74]. The high mitochondrial depolarization is not a favorable phenomenon; however, the influx of cations is balanced, e.g., by the outflow of protons in the respiratory chain [73,75]. Maintaining proper ion homeostasis prevents osmotic changes in the volume of mitochondria. Additionally, the influx of K^+^ influences ATP synthesis and favors mitochondrial respiration [76]. Perhaps this multidimensional regulation may be one of the elements of the complex phenomenon of cytoprotection. The above observations were confirmed, *inter alia*, for NS11021, a well-known mitoBK_Ca_ channel activator. Activation of this channel by NS11021 in the mitochondria of the amoeba *Dictyostelium discoideum* caused depolarization of the IMM, increase in respiration and a significant reduction in oxidative stress [54,55]. Similar results were previously obtained using NS11021 and NS1619 in the endothelial cell line EA.hy926. These effects were abolished by paxilline, the selective inhibitor of mitoBK_Ca_ [20]. In our research, we found that LUT depolarized the inner membrane in mitochondria isolated from rat hearts, and this effect is not observed under potassium-free conditions. This means the participation of the most likely mitoK channels, as it has already been shown that these channels are involved in it [77,78]. In endothelial cell model, however, an increase in mitochondrial respiration was noted in the DTT-treated sample, correlating with the results of patch-clamp experiments, where LUT activated the mitoBK_Ca_ channel under reduced DTT conditions, but not controls. Our results correlate with generally accepted principles as the activation of potassium channels causes the depolarization of the mitochondria and an increase in respiration.

MitoK channels have been shown to participate in the regulation of apoptotic cell death. Changes in the potential of the IMM as a result of the modulation of channel activity control the synthesis of ROS. Unregulated, high levels of reactive oxygen species, in turn, causes the release of cytochrome c and, consequently, apoptosis. It has been experimentally proven that mitoBK_Ca_ channel activation protects the heart against cell death due to ischemia, reducing the synthesis of ROS. Interestingly, in an animal model with a deletion of the mitoBK_Ca_ channel, under the same conditions, no protection against cell damage leading to cell death was observed [73]. Moreover, a mutation within the BK_Ca_ channel resulting in the loss of its functionality contributes to the development of cerebellar ataxia, which is observed in mitochondrial dysfunction and limited cell viability [11]. The obtained results confirm the beneficial participation of the activation of the mitoBK_Ca_ in the EA.hy926 endothelium cell model. LUT was found to be non-toxic at the applied concentrations, the induction of apoptosis and necrosis was not observed. Interestingly, LUT, depending on the concentration, protected endothelial cells from death due to apoptosis caused by TNF-α *plus* cycloheximide. This is confirmed by the fact that activators of mitochondrial potassium channels, which are these flavonoids, prevent apoptotic cell death. The results can be applied to one of the canonical activators of the mitoBK_Ca_ channel—NS11021, which also does not induce apoptosis and, like LUT, protects vascular endothelial cells against TNF/CHX-induced oxidative damage [14]. More interestingly, our in vivo studies conducted in the context of myocardial protection against I/R damage additionally confirmed, for the first time, the participation of the mitoBK_Ca_ channel in the cardioprotective action of LUT. We observed a significant lowering of damage related to the rat heart after administration of LUT (100 mg/kg) 2 h before the induction of acute myocardial infarction. These effects were partially worn by pre-treatment with 10 mg/kg paxilline. The greatest damage was observed in the group treated with DMSO (vehicle). This correlates with the data published so far as LUT already protects the heart against the ischemia/reperfusion damage. Its protective action was confirmed among others by activating Pi3K/AKS [79]; through promoting the signaling of the endogenous antioxidant enzyme, peroxirredoxin II [59]; by improving SERCA2a expression via the upregulation of Sp1 [35]; through downregulation of NO production [56] or by Sirt1/NLRP3/NF-κB pathway regulation [80]. Thus, LUT protects the heart by acting through multiple signaling pathways, one of which, as we now know, is the mitoBK_Ca_ channel, but the overall cardioprotective effect is ascribable to the interaction of LUT with a plethora of targets.

Moreover, in silico studies confirmed that LUT has an effective ability to bind the mitoBK_Ca_ channel in specific location leading to its activation. It was previously proved for other flavonoids, such as quercetin, that bounds the channel with a similar affinity to LUT, activating it and showing cytoprotective properties [22,51,81].

## 6. Conclusions

From the analysis of the literature, it emerges that LUT has a poor bioavailability—in animals as well as in humans—for the extensive first-pass metabolism by phase II enzymes. Indeed, the plasmatic peak was reached 30–40 min after oral administration, and 3 h later the main metabolites were the glucuro- and sulpho-conjugates; finally, elimination half-life (t1/2) was quantified as 2–3 h [82]. Although the low bioavailability is one of the major limitations to the therapeutic use of LUT, as well as of flavonoids, different delivery strategies, including lipid carriers and nanoformulations, have been developed and investigated in order to improve it and enable the clinical use of dietary supplements containing LUT. In any case, we can conclude that a new putative mechanism through which this flavonoid provides cell protection has been brought to light. Indeed, luteolin, as a cardioprotective substance of natural origin, activates the mitoBK_Ca_ channel, both in cardiomyocytes and in vascular endothelial cells. 

## Data Availability

Data is contained within the article.

## References

[1] O’Rourke B., Cortassa S., Aon M.A. (2005). Mitochondrial ion channels: Gatekeepers of life and death. Physiology.

[2] Goswami S.K., Ponnalagu D., Hussain A.T., Shah K., Karekar P., Gururaja Rao S., Meredith A.L., Khan M., Singh H. (2018). Expression and Activation of BKCa Channels in Mice Protects Against Ischemia-Reperfusion Injury of Isolated Hearts by Modulating Mitochondrial Function. Front. Cardiovasc. Med..

[3] Honrath B., Krabbendam I.E., Culmsee C., Dolga A.M. (2017). Small conductance Ca(^2+^)-activated K(^+^) channels in the plasma membrane, mitochondria and the ER: Pharmacology and implications in neuronal diseases. Neurochem. Int..

[4] Peng K., Hu J., Xiao J., Dan G., Yang L., Ye F., Zou Z., Cao J., Sai Y. (2018). Mitochondrial ATP-sensitive potassium channel regulates mitochondrial dynamics to participate in neurodegeneration of Parkinson’s disease. Biochim. Biophys. Acta Mol. Basis Dis..

[5] Garlid K.D. (2000). Opening mitochondrial K(ATP) in the heart—What happens, and what does not happen. Basic Res. Cardiol..

[6] Soltysinska E., Bentzen B.H., Barthmes M., Hattel H., Thrush A.B., Harper M.E., Qvortrup K., Larsen F.J., Schiffer T.A., Losa-Reyna J. (2014). KCNMA1 encoded cardiac BK channels afford protection against ischemia-reperfusion injury. PLoS ONE.

[7] Borchert G.H., Hlaváčková M., Kolář F. (2013). Pharmacological activation of mitochondrial BK(Ca) channels protects isolated cardiomyocytes against simulated reperfusion-induced injury. Exp. Biol. Med..

[8] Szewczyk A., Kajma A., Malinska D., Wrzosek A., Bednarczyk P., Zabłocka B., Dołowy K. (2010). Pharmacology of mitochondrial potassium channels: Dark side of the field. FEBS Lett..

[9] Wrzosek A., Augustynek B., Żochowska M., Szewczyk A. (2020). Mitochondrial Potassium Channels as Druggable Targets. Biomolecules.

[10] Wrzosek A. (2014). The potassium channel opener NS1619 modulates calcium homeostasis in muscle cells by inhibiting SERCA. Cell Calcium.

[11] Du X., Carvalho-de-Souza J.L., Wei C., Carrasquel-Ursulaez W., Lorenzo Y., Gonzalez N., Kubota T., Staisch J., Hain T., Petrossian N. (2020). Loss-of-function BK channel mutation causes impaired mitochondria and progressive cerebellar ataxia. Proc. Natl. Acad. Sci. USA.

[12] Toczylowska-Maminska R., Olszewska A., Laskowski M., Bednarczyk P., Skowronek K., Szewczyk A. (2014). Potassium channel in the mitochondria of human keratinocytes. J. Investig. Dermatol..

[13] Gáspár T., Domoki F., Lenti L., Katakam P.V., Snipes J.A., Bari F., Busija D.W. (2009). Immediate neuronal preconditioning by NS1619. Brain Res..

[14] Kicinska A., Kampa R.P., Daniluk J., Sek A., Jarmuszkiewicz W., Szewczyk A., Bednarczyk P. (2020). Regulation of the Mitochondrial BKCa Channel by the Citrus Flavonoid Naringenin as a Potential Means of Preventing Cell Damage. Molecules.

[15] Siemen D., Loupatatzis C., Borecky J., Gulbins E., Lang F. (1999). Ca^2+^-activated K channel of the BK-type in the inner mitochondrial membrane of a human glioma cell line. Biochem. Biophys. Res. Commun..

[16] Sato T., Saito T., Saegusa N., Nakaya H. (2005). Mitochondrial Ca^2+^-activated K^+^ channels in cardiac myocytes: A mechanism of the cardioprotective effect and modulation by protein kinase A. Circulation.

[17] Kicinska A., Augustynek B., Kulawiak B., Jarmuszkiewicz W., Szewczyk A., Bednarczyk P. (2016). A large-conductance calcium-regulated K^+^ channel in human dermal fibroblast mitochondria. Biochem. J..

[18] Skalska J., Piwonska M., Wyroba E., Surmacz L., Wieczorek R., Koszela-Piotrowska I., Zielinska J., Bednarczyk P., Dolowy K., Wilczynski G.M. (2008). A novel potassium channel in skeletal muscle mitochondria. Biochim. Biophys. Acta-Bioenerg..

[19] Sek A., Kampa R.P., Kulawiak B., Szewczyk A., Bednarczyk P. (2021). Identification of the Large-Conductance Ca(^2+^)-Regulated Potassium Channel in Mitochondria of Human Bronchial Epithelial Cells. Molecules.

[20] Bednarczyk P., Koziel A., Jarmuszkiewicz W., Szewczyk A. (2013). Large-conductance Ca(2)(^+^)-activated potassium channel in mitochondria of endothelial EA.hy926 cells. Am. J. Physiol. Heart Circ. Physiol..

[21] Stowe D.F., Yang M., Heisner J.S., Camara A.K.S. (2017). Endogenous and Agonist-induced Opening of Mitochondrial Big Versus Small Ca^2+^-sensitive K^+^ Channels on Cardiac Cell and Mitochondrial Protection. J. Cardiovasc. Pharmacol..

[22] Kampa R.P., Gliździńska A., Szewczyk A., Bednarczyk P., Filipek S. (2022). Flavonoid quercetin abolish paxilline inhibition of the mitochondrial BKCa channel. Mitochondrion.

[23] Szabo I., Zoratti M. (2014). Mitochondrial channels: Ion fluxes and more. Physiol. Rev..

[24] Kicinska A., Jarmuszkiewicz W. (2020). Flavonoids and Mitochondria: Activation of Cytoprotective Pathways?. Molecules.

[25] Duluc L., Soleti R., Clere N., Andriantsitohaina R., Simard G. (2012). Mitochondria as potential targets of flavonoids: Focus on adipocytes and endothelial cells. Curr. Med. Chem..

[26] Testai L. (2015). Flavonoids and mitochondrial pharmacology: A new paradigm for cardioprotection. Life Sci..

[27] Schroeter H., Heiss C., Spencer J.P., Keen C.L., Lupton J.R., Schmitz H.H. (2010). Recommending flavanols and procyanidins for cardiovascular health: Current knowledge and future needs. Mol. Asp. Med..

[28] Testai L., Martelli A., Cristofaro M., Breschi M.C., Calderone V. (2013). Cardioprotective effects of different flavonoids against myocardial ischaemia/reperfusion injury in Langendorff-perfused rat hearts. J. Pharm. Pharmacol..

[29] Chen Z., Kong S., Song F., Li L., Jiang H. (2012). Pharmacokinetic study of luteolin, apigenin, chrysoeriol and diosmetin after oral administration of Flos Chrysanthemi extract in rats. Fitoterapia.

[30] Lim S.H., Jung S.K., Byun S., Lee E.J., Hwang J.A., Seo S.G., Kim Y.A., Yu J.G., Lee K.W., Lee H.J. (2013). Luteolin suppresses UVB-induced photoageing by targeting JNK1 and p90RSK2. J. Cell. Mol. Med..

[31] Imran M., Rauf A., Abu-Izneid T., Nadeem M., Shariati M.A., Khan I.A., Imran A., Orhan I.E., Rizwan M., Atif M. (2019). Luteolin, a flavonoid, as an anticancer agent: A review. Biomed. Pharmacother..

[32] Chen C.Y., Peng W.H., Wu L.C., Wu C.C., Hsu S.L. (2010). Luteolin ameliorates experimental lung fibrosis both in vivo and in vitro: Implications for therapy of lung fibrosis. J. Agric. Food Chem..

[33] Domitrović R., Jakovac H., Tomac J., Šain I. (2009). Liver fibrosis in mice induced by carbon tetrachloride and its reversion by luteolin. Toxicol. Appl. Pharmacol..

[34] Li L., Luo W., Qian Y., Zhu W., Qian J., Li J., Jin Y., Xu X., Liang G. (2019). Luteolin protects against diabetic cardiomyopathy by inhibiting NF-κB-mediated inflammation and activating the Nrf2-mediated antioxidant responses. Phytomedicine.

[35] Hu Y., Zhang C., Zhu H., Wang S., Zhou Y., Zhao J., Xia Y., Li D. (2020). Luteolin modulates SERCA2a via Sp1 upregulation to attenuate myocardial ischemia/reperfusion injury in mice. Sci. Rep..

[36] Xu T., Li D., Jiang D. (2012). Targeting cell signaling and apoptotic pathways by luteolin: Cardioprotective role in rat cardiomyocytes following ischemia/reperfusion. Nutrients.

[37] Bian C., Xu T., Zhu H., Pan D., Liu Y., Luo Y., Wu P., Li D. (2015). Luteolin Inhibits Ischemia/Reperfusion-Induced Myocardial Injury in Rats via Downregulation of microRNA-208b-3p. PLoS ONE.

[38] Ferroni P., Basili S., Paoletti V., Davì G. (2006). Endothelial dysfunction and oxidative stress in arterial hypertension. Nutr. Metab. Cardiovasc. Dis..

[39] Konukoglu D., Uzun H. (2017). Endothelial Dysfunction and Hypertension. Adv. Exp. Med. Biol..

[40] Edgell C.J., McDonald C.C., Graham J.B. (1983). Permanent cell line expressing human factor VIII-related antigen established by hybridization. Proc. Natl. Acad. Sci. USA.

[41] Calderone V., Testai L., Martelli A., Rapposelli S., Digiacomo M., Balsamo A., Breschi M.C. (2010). Anti-ischemic properties of a new spiro-cyclic benzopyran activator of the cardiac mito-KATP channel. Biochem. Pharmacol..

[42] Testai L., Martelli A., Marino A., D’Antongiovanni V., Ciregia F., Giusti L., Lucacchini A., Chericoni S., Breschi M.C., Calderone V. (2013). The activation of mitochondrial BK potassium channels contributes to the protective effects of naringenin against myocardial ischemia/reperfusion injury. Biochem. Pharmacol..

[43] Bednarczyk P., Wieckowski M.R., Broszkiewicz M., Skowronek K., Siemen D., Szewczyk A. (2013). Putative Structural and Functional Coupling of the Mitochondrial BKCa Channel to the Respiratory Chain. PLoS ONE.

[44] Kampa R.P., Kicinska A., Jarmuszkiewicz W., Pasikowska-Piwko M., Dolegowska B., Debowska R., Szewczyk A., Bednarczyk P. (2019). Naringenin as an opener of mitochondrial potassium channels in dermal fibroblasts. Exp. Dermatol..

[45] Bednarczyk P., Kampa R.P., Gałecka S., Sęk A., Walewska A., Koprowski P. (2021). Patch-Clamp Recording of the Activity of Ion Channels in the Inner Mitochondrial Membrane. Methods Mol. Biol..

[46] Chu Y.H., Chen S.Y., Hsieh Y.L., Teng Y.H., Cheng Y.J. (2018). Low-level laser therapy prevents endothelial cells from TNF-alpha/cycloheximide-induced apoptosis. Lasers Med. Sci..

[47] Babu D., Soenen S.J., Raemdonck K., Leclercq G., De Backer O., Motterlini R., Lefebvre R.A. (2012). TNF-α/cycloheximide-induced oxidative stress and apoptosis in murine intestinal epithelial MODE-K cells. Curr. Pharm. Des..

[48] Tao X., MacKinnon R. (2019). Molecular structures of the human Slo1 K^+^ channel in complex with β4. eLife.

[49] Paventi G., Soldovieri M.V., Servettini I., Barrese V., Miceli F., Sisalli M.J., Ambrosino P., Mosca I., Vinciguerra I., Testai L. (2022). Kv7.4 channels regulate potassium permeability in neuronal mitochondria. Biochem. Pharmacol..

[50] Nicholls D.G., Crompton M. (1980). Mitochondrial calcium transport. FEBS Lett..

[51] Kampa R.P., Sek A., Szewczyk A., Bednarczyk P. (2021). Cytoprotective effects of the flavonoid quercetin by activating mitochondrial BKCa channels in endothelial cells. Biomed. Pharmacother..

[52] Hudson B.C., Cox J.O., Seashols-Williams S.J., Dawson Cruz T. (2021). The effects of dithiothreitol (DTT) on fluorescent qPCR dyes. J. Forensic Sci..

[53] Ko J.H., Ibrahim M.A., Park W.S., Ko E.A., Kim N., Warda M., Lim I., Bang H., Han J. (2009). Cloning of large-conductance Ca(^2+^)-activated K(^+^) channel alpha-subunits in mouse cardiomyocytes. Biochem. Biophys. Res. Commun..

[54] Krabbendam I.E., Honrath B., Culmsee C., Dolga A.M. (2018). Mitochondrial Ca(^2+^)-activated K(^+^) channels and their role in cell life and death pathways. Cell Calcium.

[55] Laskowski M., Kicinska A., Szewczyk A., Jarmuszkiewicz W. (2015). Mitochondrial large-conductance potassium channel from Dictyostelium discoideum. Int. J. Biochem. Cell Biol..

[56] Liao P.H., Hung L.M., Chen Y.H., Kuan Y.H., Zhang F.B., Lin R.H., Shih H.C., Tsai S.K., Huang S.S. (2011). Cardioprotective effects of luteolin during ischemia-reperfusion injury in rats. Circ. J..

[57] Zhang R.Q., Li D.Y., Xu T.D., Zhu S.S., Pan H.J., Fang F., Wu X., Sun H. (2017). Antioxidative effect of luteolin pretreatment on simulated ischemia/reperfusion injury in cardiomyocyte and perfused rat heart. Chin. J. Integr. Med..

[58] Luo Y., Shang P., Li D. (2017). Luteolin: A Flavonoid that Has Multiple Cardio-Protective Effects and Its Molecular Mechanisms. Front. Pharmacol..

[59] Wei B., Lin Q., Ji Y.G., Zhao Y.C., Ding L.N., Zhou W.J., Zhang L.H., Gao C.Y., Zhao W. (2018). Luteolin ameliorates rat myocardial ischaemia-reperfusion injury through activation of peroxiredoxin II. Br. J. Pharmacol..

[60] Calderone V., Chericoni S., Martinelli C., Testai L., Nardi A., Morelli I., Breschi M.C., Martinotti E. (2004). Vasorelaxing effects of flavonoids: Investigation on the possible involvement of potassium channels. Naunyn-Schmiedebergs Arch. Pharmacol..

[61] Jiang H., Xia Q., Wang X., Song J., Bruce I.C. (2005). Luteolin induces vasorelaxion in rat thoracic aorta via calcium and potassium channels. Pharmazie.

[62] Seo A., Jackson J.L., Schuster J.V., Vardar-Ulu D. (2013). Using UV-absorbance of intrinsic dithiothreitol (DTT) during RP-HPLC as a measure of experimental redox potential in vitro. Anal. Bioanal. Chem..

[63] Liu T.H., Yaghmour M.A., Lee M.H., Gradziel T.M., Leveau J.H.J., Bostock R.M. (2020). An roGFP2-Based Bacterial Bioreporter for Redox Sensing of Plant Surfaces. Phytopathology.

[64] Rotko D., Kunz W.S., Szewczyk A., Kulawiak B. (2020). Signaling pathways targeting mitochondrial potassium channels. Int. J. Biochem. Cell Biol..

[65] Olschewski A., Weir E.K. (2015). Redox regulation of ion channels in the pulmonary circulation. Antioxid. Redox Signal..

[66] Messias Sandes J., Nascimento Moura D.M., Divina da Silva Santiago M., Barbosa de Lima G., Cabral Filho P.E., da Cunha Gonçalves de Albuquerque S., de Paiva Cavalcanti M., Fontes A., Bressan Queiroz Figueiredo R.C. (2019). The effects of endoplasmic reticulum stressors, tunicamycin and dithiothreitol on Trypanosoma cruzi. Exp. Cell Res..

[67] Ługowski M., Saczko J., Kulbacka J., Banaś T. (2011). Reactive oxygen and nitrogen species. Pol. Merkur. Lek..

[68] Larosa V., Remacle C. (2018). Insights into the respiratory chain and oxidative stress. Biosci. Rep..

[69] Zorov D.B., Juhaszova M., Sollott S.J. (2014). Mitochondrial reactive oxygen species (ROS) and ROS-induced ROS release. Physiol. Rev..

[70] Testai L., Da Pozzo E., Piano I., Pistelli L., Gargini C., Breschi M.C., Braca A., Martini C., Martelli A., Calderone V. (2017). The Citrus Flavanone Naringenin Produces Cardioprotective Effects in Hearts from 1 Year Old Rat, through Activation of mitoBK Channels. Front. Pharmacol..

[71] Walewska A., Krajewska M., Stefanowska A., Buta A., Bilewicz R., Krysiński P., Bednarczyk P., Koprowski P., Szewczyk A. (2022). Methods of Measuring Mitochondrial Potassium Channels: A Critical Assessment. Int. J. Mol. Sci..

[72] Walewska A., Kulawiak B., Szewczyk A., Koprowski P. (2018). Mechanosensitivity of mitochondrial large-conductance calcium-activated potassium channels. Biochim. Biophys. Acta Bioenerg..

[73] Checchetto V., Azzolini M., Peruzzo R., Capitanio P., Leanza L. (2018). Mitochondrial potassium channels in cell death. Biochem. Biophys. Res. Commun..

[74] Szewczyk A., Jarmuszkiewicz W., Kunz W.S. (2009). Mitochondrial potassium channels. IUBMB Life.

[75] Beavis A.D., Lu Y., Garlid K.D. (1993). On the regulation of K^+^ uniport in intact mitochondria by adenine nucleotides and nucleotide analogs. J. Biol. Chem..

[76] Heinen A., Camara A.K.S., Aldakkak M., Rhodes S.S., Riess M.L., Stowe D.F. (2007). Mitochondrial Ca^2+^-induced K^+^ influx increases respiration and enhances ROS production while maintaining membrane potential. Am. J. Physiol.-Cell Physiol..

[77] Debska G., May R., Kicińska A., Szewczyk A., Elger C.E., Kunz W.S. (2001). Potassium channel openers depolarize hippocampal mitochondria. Brain Res..

[78] Testai L., Sestito S., Martelli A., Gorica E., Flori L., Calderone V., Rapposelli S. (2021). Synthesis and pharmacological characterization of mitochondrial K(ATP) channel openers with enhanced mitochondriotropic effects. Bioorg. Chem..

[79] Sun D., Huang J., Zhang Z., Gao H., Li J., Shen M., Cao F., Wang H. (2012). Luteolin limits infarct size and improves cardiac function after myocardium ischemia/reperfusion injury in diabetic rats. PLoS ONE.

[80] Zhao L., Zhou Z., Zhu C., Fu Z., Yu D. (2020). Luteolin alleviates myocardial ischemia reperfusion injury in rats via Siti1/NLRP3/NF-κB pathway. Int. Immunopharmacol..

[81] Fusi F., Trezza A., Tramaglino M., Sgaragli G., Saponara S., Spiga O. (2020). The beneficial health effects of flavonoids on the cardiovascular system: Focus on K(^+^) channels. Pharmacol. Res..

[82] Taheri Y., Sharifi-Rad J., Antika G., Yılmaz Y.B., Tumer T.B., Abuhamdah S., Chandra S., Saklani S., Kılıç C.S., Sestito S. (2021). Paving Luteolin Therapeutic Potentialities and Agro-Food-Pharma Applications: Emphasis on In Vivo Pharmacological Effects and Bioavailability Traits. Oxid. Med. Cell Longev..

